# Effects of a 12-Month Intensive Lifestyle Monitoring Program in Predominantly Overweight/Obese Arab Adults with Prediabetes

**DOI:** 10.3390/nu12020464

**Published:** 2020-02-12

**Authors:** Kaiser Wani, Hanan Alfawaz, Abdullah M. Alnaami, Shaun Sabico, Malak Nawaz Khan Khattak, Omar Al-Attas, Majed S. Alokail, Mohammed Alharbi, George Chrousos, Sudhesh Kumar, Nasser M. Al-Daghri

**Affiliations:** 1 Biochemistry Department, Chair for Biomarkers of Chronic Diseases, King Saud University, Riyadh 11451, Saudi Arabia; wani.kaiser@gmail.com (K.W.); aalnaami@yahoo.com (A.M.A.); eaglescout01@yahoo.com (S.S.); malaknawaz@yahoo.com (M.N.K.K.); omrattas@ksu.edu.sa (O.A.-A.); msa85@yahoo.co.uk (M.S.A.); 2 Department of Food Science and Nutrition, College of Food Science and Agriculture, King Saud University, Riyadh 11451, Saudi Arabia; halfawaz@ksu.edu.sa; 3 Diabetes Centres and Units Administration, Ministry of Health, Riyadh 11176, Saudi Arabia; malharbi24@yahoo.com; 4 First Department of Pediatrics, Medical School, National and Kapodistrian University of Athens, 15772 Athens, Greece; chrousos@gmail.com; 5 Division of Biomedical Sciences, Warwick Medical School, University of Warwick, Coventry CV4 7HL, UK; Sudhesh.Kumar@warwick.ac.uk

**Keywords:** lifestyle intervention, type 2 diabetes, impaired glucose regulation, fasting blood glucose, physical activity, dietary modifications

## Abstract

This 12-month, randomized, controlled lifestyle intervention study was aimed at assessing the effectiveness of a lifestyle intervention in terms of (1) the reduction of at least 5% of body weight compared to baseline and (2) the percentage of participants in which fasting blood glucose (FBG) normalizes (<5.6 mmol/L) post-intervention, in predominantly overweight/obese Saudi adults with impaired fasting glucose. A total of 300 Saudi adults with prediabetes at baseline (FBG 5.6–6.9 mmol/L) were enrolled to receive either general advice (GA) or a rigorous, self-monitored, lifestyle modification program (intervention group, IG) for 12 months, focused on food choices, physical activity, and weight loss. Anthropometric and biochemical estimations were analyzed at baseline, 6, and 12 months. At baseline, 136/150 in the GA group (90.7%) and 127/150 in the IG group (84.7%) were either overweight or obese. A total of 14% (*n* = 21) of the subjects in the IG arm discontinued, compared to 8% (*n* = 12) in the GA arm. Data from completers (92% (*n* = 138) and 86% (*n*= 129) participants in GA and IG arms, respectively) were considered for the final analysis. Post-intervention, 37.2% (*n* = 48) of participants in the IG group had ≥5% weight reduction, as compared to 12.3% (*n* = 17) in the GA group (*p* < 0.01). Similarly, the percentage of participants who normalized their FBG post-intervention was 46.5% (*n* = 60) in the IG group compared to 21.7% (*n* = 30) in the GA group (*p* < 0.01). A 12-month Diabetes Prevention Program (DPP)-styled intensive lifestyle program translated effectively in decreasing weight and improving fasting glucose compared to the GA group in predominantly overweight/obese Saudi adults with prediabetes, suggesting that in the case of guided intervention programs, people are willing to participate and possibly change a sedentary lifestyle.

## 1. Introduction

The epidemic of type 2 diabetes mellitus (T2DM) continues to be unabated. The global burden of T2DM is expected to rise to 366 million in 2030 [1]. The Middle Eastern region is no different from the rest of the world in terms of high T2DM prevalence [2,3]. An estimate released by the International Diabetes Federation (IDF) in 2015 indicated that Saudi Arabia is among the top 10 countries worldwide with the highest prevalence of T2DM [4]. Key determinant factors associated with T2DM in the Arab world, aside from the genetic predisposition are obesity, rapid urbanization, and lack of physical activity [5]. The lack of an effective large-scale lifestyle modification program focusing on T2DM prevention, particularly in Saudi Arabia, has most likely exacerbated the alarming increase in the prevalence of T2DM. 

In a large-scale randomized clinical trial [Diabetes Prevention Program (DPP)] led by the National Institute of Diabetes and Digestive and Kidney Diseases [6], weight loss through caloric restriction and increased physical activity significantly delayed progression to T2DM among individuals with impaired glucose regulation (prediabetes). The aforementioned study confirmed previous landmark studies, such as the Finnish Diabetes prevention study (FDPS) [7] and the Da Qing study in China [8], in establishing the clinical importance of healthy lifestyle changes for T2DM prevention. Studies have shown that without intervention, around 5%–10% of individuals with prediabetes eventually progress to full-blown T2DM within a year [9]. Normalization of glycemic status through monitored weight reduction has been the focus of lifestyle modification programs to delay T2DM and its associated complications [10]. Educating people about T2DM in general, particularly individuals at a high risk for impaired fasting regulation, should be done at the level of primary health care centers, when the a hyperglycemic profile is first encountered [11,12]. The primary health care system is most suitable for the prevention and promotion of healthy well-being because of its easy accessibility; continuity of care through follow-up; and its usage by a large percentage of the people [13]. 

Since the findings presented by the landmark DPP lifestyle intervention trial, not much has been translated into practice, especially in the Arab region. Implementing such programs in a homogenous population, such as in Saudi Arabia, particularly in health care settings, has unique challenges. The culturally-conservative nature of the Saudi society is one impediment to deliver such lifestyle intervention programs [14]. Physical inactivity is also widespread, and in fact, according to a national survey published by the Ministry of Health in 2013, 46% and 75.1% of Saudi men and women, respectively, are either physically inactive or have low levels of physical activity [15]. These figures are higher compared to the global average of insufficient physical activity (23.4% in men and 31.7% in women) [16]. In addition, women in Saudi Arabia should wear full length outer garments in public and are reluctant to engage in outdoor physical activities [17]. Another important barrier is the lack of funds allocated for such lifestyle interventions at these settings [18].

For a large-scale lifestyle intervention program in Saudi Arabia to work, it is necessary to remove or minimize the barriers mentioned above. Thus far, data reporting on such interventions in this homogenous society is scarce. Efforts are being made by the Chair of Biomarkers in Chronic Diseases (CBCD) at King Saud University (KSU) in collaboration with King Salman Hospital (KSH), Riyadh, Saudi Arabia to establish lifestyle intervention programs focusing on overweight/obese adults with impaired fasting glucose. Patient-centered education on the risks of having impaired fasting glucose is critical in encouraging behavioral changes and to effectively counsel participants on lifestyle modifications. The program included DPP-styled self-monitoring of blood glucose and weight [19] along with tailored interventions, taking into consideration cultural contexts [20]. 

To lay the groundwork for a large-scale DPP in Saudi Arabia, the investigators at CBCD, KSU, along with the clinicians at KSH, devised a DPP-based lifestyle modification program focused on overweight/obese Saudi adults with impaired fasting glucose. The aim of the study was to evaluate the effectiveness of the intervention, with weight reduction (≥5%) and normalization of FBG (<5.6 mmol/L) as primary outcomes.

## 2. Materials and Methods

This 12-month two-arm randomized controlled (1:1) lifestyle intervention study was conducted by CBCD, KSU in collaboration with the diabetes center at KSH from April 2013 to March 2017. The protocol was approved by the Ethics Committee of the College of Science, KSU (reference# 8/25/220355) and was conducted in accordance with the ethical standards set by the Helsinki Declaration of 1975.

### 2.1. Study Design and Participants

In this interventional study, consenting participants were Saudi adults (≥20 years) visiting KSH and other centers for a routine overnight-fast blood withdrawal. Willing participants were enrolled if they had impaired glucose tolerance (FBG 5.6–6.9 mmol/L) [21]. The exclusion criteria were: Expatriates; pregnant women; those with established type 1 or T2DM and/or those on anti-hyperglycemic drugs; and those with chronic medical conditions, such as renal, hepatic, and cardiac complications. 

**Figure 1:**
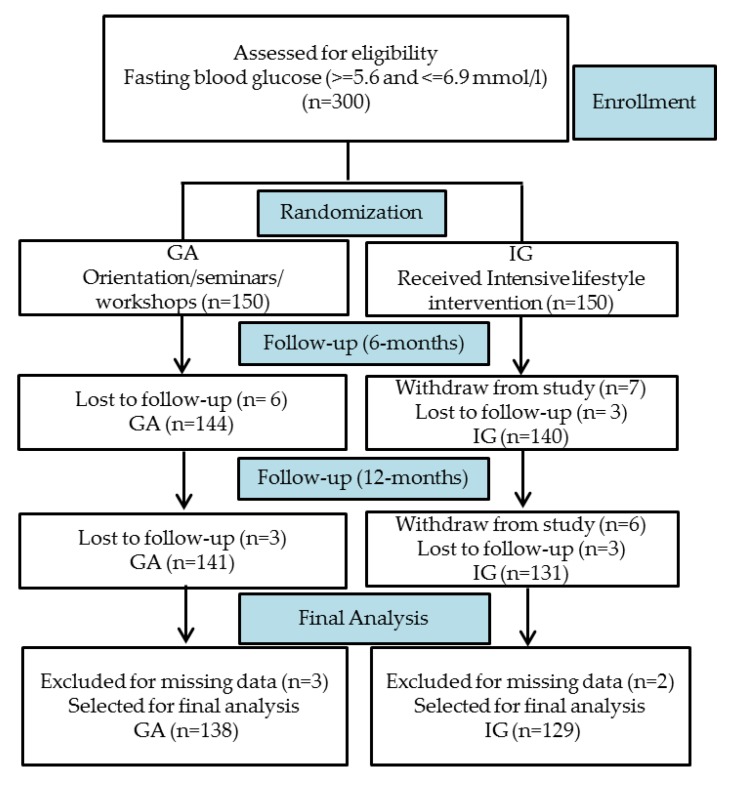
Flow chart of study participants.

Within a span of four years after the interventional program was conducted, a total of 300 predominantly overweight/obese Saudi adults with impaired fasting glucose consented to take part and were randomly (1:1) divided to receive either an intensive lifestyle modification program (Intervention group, IG = 150) or general advise at the time of recruitment (General Advise group, GA = 150). Out of 300 participants recruited, 138 participants in GA and 129 participants in IG completed the program. A total of 33 participants dropped out at different stages of follow-up due to various reasons, such as loss to follow-up, withdrawal from participation due to a delay in test results, transferring to a different city, among other reasons. A flow chart is provided in Figure 1.

### 2.2. Intervention

Both groups (GA and IG) had an orientation session conducted by a dietician and a physician at the respective study centers. Participants were educated about the risks of prediabetes, overweight/obesity and associated complications, the current scenario of diabetes worldwide and in Saudi Arabia, as well as benefits of modifying lifestyle through dietary changes and increased in physical activity. Pamphlets and booklets containing information on healthy food and lifestyle choices, nutritional components of foods, “Healthy Eating Plate (HEP)”, sedentary behavior, and the benefits of physical activity, were explained and distributed to all participants. The session lasted an hour and was conducted for a group of 3–10 newly recruited participants. Aside from the orientation session, seminars and workshops on related topics presided by the investigators were conducted in each center every four months, and all participants were invited to attend.

Participants in the GA group received only the intervention, as described above. However, participants in the IG group were additionally given an intensive lifestyle modification support, as published earlier [22,23]. The interventions given to the groups are briefly summarized in Table 1.

### 2.3. Clinical and Biochemical Characteristics

Clinical characteristics were measured by trained nurses at their respective centers at the time of recruitment (baseline), and at 6-months and 12-months of intervention. These included anthropometric measurements such as height (cm), weight (kg), waist and hip circumferences (cm), and blood pressure (mmHg). A standard weighing scale (Digital Pearson, ADAM equipment Inc., Oxford, CT, USA) was used to measure body weight and height of the participants. Body mass index (BMI) was calculated using the standard formula as kg/m^2^. A standard measuring tape was used to assess waist and hip circumferences. A conventional mercurial sphygmomanometer was used to measure systolic and diastolic blood pressure. 

An overnight fasting blood sample was collected in a red-top vacutainer from each participant for each time-point and centrifuged using a standard tabletop centrifuge for the separation of serum. Serum samples collected were aliquoted in multiple 1.5 μL Eppendorf tubes and transported immediately to CBCD, KSU for biochemical estimations of fasting glucose, insulin, and lipid profile using an automated biochemical analyzer (Konelab 20, Thermo-Fischer, Espoo, Finland). A standard glucose-oxidase-peroxidase (GOD-POD) method was used to assess fasting glucose (catalogue # 981379 by Thermo-Fischer). A commercial kit for Multiplex (Luminexcorp, Austin, TX, USA) was used to assess fasting insulin levels. Coefficients of variation (CV) for these estimations were ≤5%, ≤3.5%, ≤4%, ≤4%, and ≤4.5% for glucose, total cholesterol, HDL-cholesterol, triglycerides, and insulin tests, respectively.

### 2.4. Statistical Analysis

The sample size calculation was done using G*power 3.1.9.4 software [24]. For a two-tailed paired t-test with effect size 0.3, α = 0.05, and power 0.90; a sample size of at least 119 subjects were required per group. The clinical and biochemical data collected for the three time-points for each participant was compiled and analyzed using SPSS version 23. The data was checked for normality using the Kolmogorov–Smirnov test. The data at baseline was presented as mean ± standard deviation for normally distributed variables, median (Quartile 1, Quartile 3) for continuous non-normally distributed variables, and *n* (%) for categorical variables. Further analysis was done using data of participants who completed the entire program

Intervention effects giving the adjusted mean difference within groups were calculated using repeated measures analysis of co-variance (ANCOVA) with total cholesterol, HDL-cholesterol, and insulin as covariates. A 5% weight loss from baseline was the target goal, as reported in earlier interventions [25,26]. The number of participants (% group) who showed a mean change of at least 5% from the baseline body weight and fasting glucose was counted and the proportions were tested between study arms using the Chi-Square test. A *p* < 0.05 was considered significant.

## 3. Results

### 3.1. General Characteristics of the Study Participants at Recruitment

Table 2 shows the anthropometric, physiological, and biochemical characteristics of the study participants at the time of recruitment. Most participants (64% in GA group and 66.6% in IG group) were within 40–73 years (72% and 66.6% were females in GA and IG group, respectively) In addition, the majority of the participants were either overweight or obese (90.7% and 84.7% in GA and IG groups, respectively). At the time of recruitment, all anthropometric characteristics of the two groups were comparable, including fasting glucose and triglyceride levels. However, the two groups were slightly different in terms of their circulating levels of total cholesterol; HDL-cholesterol; and insulin. 

Baseline characteristics were also analyzed according to those who completed the study and those who discontinued (Supplementary Table S1); and according to sex (Supplementary Table S2). The improvements in variables, which had a sex-specific cut-off, were also analyzed over time, separately in females (Supplementary Table S3) and males (Supplementary Table S4).

### 3.2. Clinical Characteristics of the Study Groups’ Over Time

Table 3 shows the clinical parameters of the study participants at baseline, 6-months, and 12-months. It also shows the intervention effects in terms of adjusted mean differences at 6-months and 12-months. Baseline total-cholesterol, HDL-cholesterol, and insulin were controlled to calculate mean differences. This analysis was done for subjects who completed the program (138/150 in GA and 129/150 in the IG group). Weight reduced significantly in the IG group only. Glycemic indices also improved only in the IG group and not in the GA group. In the IG group, fasting glucose improved at 6-months post-intervention and further improved significantly at 12-months. No significant improvement in fasting glucose was observed in the GA group, although it slightly decreased post 12-months of intervention. No significant improvements over time were seen in the lipid levels of both groups. 

### 3.3. Percentage Change in Body Weight and Fasting Glucose from Baseline to End of Study

Table 4 gives the data on the accomplishment of the primary objectives of this study in terms of the reduction in body weight and fasting glucose post-intervention in the two study groups. The data is given in percentage change post-intervention in body weight and fasting glucose, and also percentage of subjects who normalized their fasting glucose post-intervention (FG < 5.6 mmol/L). 

## 4. Discussion

A significant rise in the global economic burden of T2DM is seen worldwide. In the US alone, the medical costs related to diabetes rose over 33% from $245 billion in 2012 to $327 billion in 2017 [27]. According to IDF Diabetes Atlas, 8th edition (2017), the health expenditure due to diabetes in the Middle East and North Africa (MENA) region was $20 billion in 2017, and is expected to increase to $37 billion by 2045 [28]. The blame rests not only on the increased cost for every individual with diabetes, but also on the overall increase in the incidence of diabetes worldwide [29]. The importance of strategies to reduce the prevalence of T2DM, such as prevention practices in prediabetes and/or obese individuals, are therefore highlighted in the recent past as an effective measure to control the cost of T2DM. In Saudi Arabia, such lifestyle modification programs focused on this high-risk group are limited. Hence, this 12-month, DPP-style program was carried out in Saudi adults with prediabetes to determine its effectiveness in terms of glycemic control. The results of this study showed statistically significant changes in body weight, BMI, and glycemic indices post-intervention in favor of the IG. 

Excess body-weight (particularly obesity) is regarded as a common risk factor for diabetes [30]. The relationship between the two is so interlinked that it has led to the emergence of a new epidemic called “diabesity” [31]. In the present study, the majority of the participants were either overweight or obese in GA (91%) and IG (85%) groups, with a small percentage of participants under the lean category, but nevertheless with an excess amount of visceral adiposity. It has generally been accepted that weight reduction in high-risk individuals leads to the prevention and delay of T2DM progression [25,32,33]. The DPP, in a 3.2 year follow-up analysis, reported a reduction of 16% in the incidence of diabetes per one kg loss in weight [26]. In our lifestyle interventional program, the significant improvement in the glycemic indices, such as the average reduction of 0.47 mmol/L from baseline fasting glucose levels in the IG arm, may be because of the significant reduction in bodyweight. An average reduction of 1.96 kg from baseline bodyweight was seen in the IG arm compared to an average increase of 0.57 kg in the GA arm. This weight loss in the intervention arm is comparable to some of the figures reported earlier in some lifestyle interventions conducted worldwide, with the examples being an average change of −1.75 kg [34], −2.18 kg [32], −2.30 kg [35], and −2.59 kg [36]; however, it is still lower than the average weight loss of 5.6kg and 4.5kg reported in two of the biggest lifestyle intervention studies on individuals with prediabetes: the DPP [25] and the FDPS, respectively [24]. The differences in the nature of the lifestyle intervention program in terms of the intensive support or the self-monitoring methodology (duration, extent of the follow-ups conducted, culture-specific hindrances, adherence, etc.) would have contributed to the differences seen in these studies and the one done in our investigation.

In our study, 37% (*n* = 48) of the participants in the IG group managed to reduce their body weight over time by at least 5% as compared to 12.3% (*n* = 17) observed in the GA group. Studies have reported improved β-cell function and improved insulin sensitivity in the liver and skeletal muscle cells as a consequence of 5% weight loss [37]. Given a strong relationship between T2DM risk and weight gain, the focus of a suitable intervention program on obese individuals with prediabetes should be the prevention of further weight gain, even if there was no weight loss. In this study, 70.5% (*n* = 91) of subjects in the IG group displayed no further weight gain post-intervention compared to 18.1% (*n* = 25) of subjects in the GA arm, which depicted the effectiveness of the intervention program implemented for weight control compared to the standard care. 

Though weight loss by increased physical activity has its importance, changing unhealthy eating habits is also believed to play a huge impact. Dietary modifications, such as reducing total fat intake (especially saturated fats), choosing better sources of carbohydrates, a higher intake of foods rich in fiber content, a preference for whole grains and natural foods over processed foods, etc., are all pivotal in achieving the target goals of our lifestyle intervention. Dietary patterns such as the Mediterranean diet are high in fiber-rich foods like fruits, vegetables, whole grains, olive oil as the main fat source, etc., and have been observed to be protective against diabetes; while Western diets rich in refined cereals, processed foods, animal fat as main fat source, etc., have been found to be associated with higher risk [38]. In this study, we were not able to record the changes in the dietary patterns overtime; however, in a similar, yet smaller, pilot study published earlier, we showed that weight reduction was due to significant caloric restriction, as observed in reduced macro/micronutrient intakes over time [39]. 

One of the challenging aspects is that people with prediabetes present no apparent clinical symptoms. In one report from the Center for Disease Control and Prevention (CDC) [40], 33.9% of U.S. adults had prediabetes in 2015 but only 11.6% were aware and had symptoms. This stresses the need to target normoglycemic but high-risk populations, such as overweight/obese individuals. In the present study, we examined the incidence of reversion to a normoglycemic state and found that 46.5% (*n* = 60) of the participants in the intervention arm achieved this in comparison to 21.7% (*n* = 30) in the standard care arm. This is consistent with the data presented by Xu et al. [34] and Moore et al. [36], who respectively reported this to be 39% and 43% in the intervention arm and 7.5% and 26% in the control group. 

Weight loss can generally be seen as an important component that enhances the metabolic profile, and hence, reversal to a normoglycemic state. Other components independent of weight loss, such as healthy eating and increased physical activity, have previously been demonstrated to have beneficial effects on insulin sensitivity [41]. Reversion to normoglycemia, even if for a transient period, has been shown to be better than remaining at the prediabetes state. Data from the Diabetes Prevention Program Outcome Study (DPPOS) revealed that diabetes risk was 56% higher in participants who remained in the prediabetes state than in participants who transiently returned to normal glucose levels [42]. The greater regression to a normoglycemic state in the IG group in this study reflects the effectiveness of this program in preserving β-cell function of participants and the preservation of this function was found to relevant in diabetes prevention [42].

The authors acknowledge some limitations. Clinically more relevant outcome variables, such as glycated hemoglobin, were not measured and would have been a better primary outcome than weight loss and FBG. Furthermore, it would be interesting to implement such programs in a longer term and on a larger scale to have a real impact on public health in Saudi Arabia. Since this program was conducted in adults with prediabetes, the findings cannot be generalized for other high-risk populations, such as obese children or those at risk for gestational diabetes. Additionally, the study was not designed to assess the actual dietary modifications or changes in dietary patterns, nor changes in physical activity levels post-intervention. Despite these limitations, however, this self-monitoring-based lifestyle intervention conducted in a Saudi healthcare setting showed significant reversal to normoglycemia and significant weight loss in Saudi adults with impaired fasting glucose. Findings have clinical merit in a region where the conduct of these studies are limited. Further investigations that include the economic impact of such interventions are warranted.

## 5. Conclusions

A 12-month DPP-styled intensive lifestyle program implemented in a primary health care setting was effective in decreasing weight and improving glycemic status in predominantly overweight/obese Saudi adults with prediabetes. Findings from this study should further be evaluated in the context of the long-term maintenance of a normoglycemia state.

## Figures and Tables

**Table 1:** Intervention given to study groups.

Lifestyle Intervention	GA Group	IG Group
(A) Diabetes Education	● Orientation Session at recruitment ● Distribution of pamphlets/booklets at recruitment ● Seminars/workshops every four months	● Orientation Session at recruitment ● Distribution of pamphlets/booklets at recruitment ● Seminars/workshops every four months
● Health effects of impaired fasting glucose ● Health effects of overweight/obesity ● Risk of developing diabetes		
(B) Education on Healthy diet		
● Healthy food and lifestyle choices ● Nutritional components of food ● Healthy Eating Plate (HEP)		
(C) Education on benefits of physical activity		
● Health effects of sedentary behavior ● Concept of wellbeing		
(D) Dietary counseling	● No	● Individual consultation with a dietician ● Follow up through phone call/email/short message service
● Assessment of food intake ● Special dietary charts ● Ways to reduce total dietary fat <30% of energy ● Ways to increase fiber consumption to 15 g/1000 Kcal		
(E) Physical activity counseling	● No	● Individual consultation with a physical therapist ● Pedometers (081565583, Patterson Medical) supplied ● Recommended at least 5000 steps/day ● Follow up through phone call/email/short message service
● Assessment of physical activity levels ● Special physical activity/exercise/yoga charts ● Concept of calorie burn ● Concept of Metabolic Equivalents (MET) ● Saudi guidelines for management of obesity		
(F) Fasting blood samples and Anthropometrics	● Baseline, 6M and 12M ● Baseline and every six months	● Baseline, 6M and 12M ● Baseline and every six months

Note: GA is “general advice group” and IG is “intervention group”. 6M and 12M are 6 and 12 months, respectively.

**Table 2:** Baseline anthropometric and biochemical characteristics of all study participants.

Parameters	GA (*n* = 150)	IG (*n* = 150)	*p* -Value
**Age group (years) ^**			
20-29	14 (9.3)	13 (8.7)	0.09
30-39	40 (26.7)	37 (24.7)	
40-49	50 (33.3)	60 (40.0)	
50-73	46 (30.7)	40 (26.6)	
**Marital Status ^**			
Married	84 (56.0)	76 (50.7)	0.11
Not married	21 (14.0)	18 (12.0)	
NA	45 (30.0)	56 (37.3)	
**Sex^**			
Male	42 (28.0)	50 (33.3)	0.12
Female	108 (72.0)	100 (66.6)	
**Nutritional status ^**			
Lean	8 (5.3)	14 (9.3)	0.25
Overweight	40 (26.7)	46 (30.7)	
Obese	96 (64.0)	81 (54.0)	
NA	6 (4.0)	9 (6.0)	
Age (years)#	43.75 ± 10.9	43.10 ± 9.4	0.61
Weight (kg)#	81.95 ± 14.3	81.26 ± 15.5	0.72
BMI (kg/m^2^)#	32.92 ± 6.0	31.71 ± 6.0	0.12
Waist (cm)#	96.96 ± 8.6	96.45 ± 13.4	0.74
Hips (cm)#	110.87 ± 9.1	111.85 ± 11.4	0.48
Systolic BP (mmHg)#	120.17 ± 13.5	121.54 ± 14.7	0.45
Diastolic BP (mmHg)#	76.46 ± 11.6	76.3 ± 11.0	0.91
Total Cholesterol (mmol/L)#	4.85 ± 1	5.29 ± 1.3	0.001
HDL-Cholesterol (mmol/L)#	1.08 ± 0.3	1.17 ± 0.4	0.03
Triglycerides (mmol/L)#	1.45 (1.1, 2.0)	1.49 (1.1, 2.0)	0.84
Glucose (mmol/L)#	6.17 ± 0.6	6.09 ± 0.4	0.22
Insulin (μU/mL)$	15.83 (12.4, 16.5)	16.59 (16.4, 16.7)	0.01

Note: Data presented as *n* (%) for categorical variables (^); Mean ± SD for continuous normal variables (#); and medians (25th percentile, 75th percentile) for continuous non-normal variables ($). The difference between groups at baseline was calculated by the independent samples t-test and Mann–Whitney U-test for Gaussian and non-Gaussian variables, respectively, and χ² test for categorical variables. *p* < 0.05 was taken as significant. NA, data not available; BP, blood pressure; HDL, high density lipoprotein.

**Table 3:** Anthropometric, glycemic, and lipid indices of participants over time.

	Groups		Intervention Effects (Adjusted Mean Change, *p*)			
Time-point	GA (*n* = 138)	IG (*n* = 129)	GA (6M vs. B)	GA (12M vs. B)	IG (6M vs. B)	IG (12M vs. B)
**Anthropometric characteristics**						
Weight (kg) #						
Baseline	82.56 ± 13.8	80.71 ± 15.7	0.47, 0.48	0.57, 0.46	−0.91, 0.06	−1.96, <0.01
6-months	83.09 ± 14.1	79.56 ± 15.6				
12-months	83.27 ± 13.7	78.01 ± 15.8				
BMI (kg/m^2^) #						
Baseline	33.13 ± 5.9	31.67 ± 6.0	0.20, 0.42	0.24, 0.44	−0.31, 0.15	−0.76, <0.01
6-months	33.36 ± 6.1	31.24 ± 6.1				
12-months	33.39 ± 5.9	30.57 ± 6.3				
**Physiological Indices**						
Systolic Blood Pressure (mmHg) #						
Baseline	120.28 ± 13.5	121.23 ± 14.6	−2.25, 0.36	−0.84, 1.0	−2.12, 0.52	−2.59, 0.08
6-months	118.30 ± 14.7	119.83 ± 17.9				
12-months	119.20 ± 15.9	118.27 ± 16.9				
Diastolic Blood Pressure (mmHg) #						
Baseline	76.70 ± 11.8	76.08 ± 10.8	−0.23, 0.96	0.27, 0.72	−0.43, 0.84	−1.46, 0.15
6-months	76.43 ± 11.9	75.57 ± 11.9				
12-months	77.07 ± 13.5	74.53 ± 12.2				
**Glycemic Indices**						
Fasting Glucose (mmol/L) #						
Baseline	6.17 ± 0.6	6.11 ± 0.4	0.07, 1.00	−0.18, 0.52	−0.38, <0.01	−0.47, <0.01
6-months	6.16 ± 1.1	5.72 ± 1.1				
12-months	5.92 ± 0.8	5.59 ± 0.8				
Insulin (μU/mL) $						
Baseline	15.78 (11.9,16.1)	16.56 (16.5,16.7)	0.02, 0.18	0.03, 0.45	0.04, 0.39	−0.03, 0.05
6-months	15.80 (10.5,15.9)	16.65 (16.6,16.7)				
12-months	15.83 (11.6,15.9)	15.99 (15.9,16.1)				
**Lipid Indices**						
Total Cholesterol (mmol/L) #						
Baseline	4.84 ± 0.9	5.25 ± 1.2	−0.06, 1.00	−0.23, 0.12	−0.19, 0.24	0.28, 0.17
6-months	4.67 ± 1.1	5.10 ± 1.1				
12-months	4.63 ± 1.1	4.94 ± 1.0				
HDL-Cholesterol (mmol/L) #						
Baseline	1.07 ± 0.3	1.17 ± 0.4	−0.10, 0.06	−0.13, 0.02	0.03, 1.00	0.05, 1.00
6-months	0.94 ± 0.4	1.19 ± 0.4				
12-months	0.97 ± 0.4	1.15 ± 0.4				
Triglycerides (mmol/L) $						
Baseline	1.47 (1.1,2.1)	1.48 (1.1,2.0)	0.02, 1.00	−0.01, 0.64	−0.04, 0.23	−0.03, 0.17
6-months	1.51 (1.1,2.1)	1.35 (1.0,1.9)				
12-months	1.43 (1.1,2.0)	1.40 (1.0,1.9)				

Note: Data presented as mean ± SD for continuous normal variables (#); and medians (25th percentile, 75th percentile) for continuous non-normal variables ($). Non-normal variables ($) were log-transformed prior to further analysis. The three time-points were baseline (B), 6-months (6M), and 12-months (12M). The intervention affects gives the adjusted mean difference within groups at follow-up compared with baseline and was calculated by repeated measures ANCOVA with Total cholesterol, HDL-C, and insulin as covariates. *p*-value < 0.05 was considered as significant.

**Table 4:** Percentage change in primary endpoints according to groups.

Category	% Change at End of Study	GA *n* (%)	IG *n* (%)	*p*
**Weight**				
1	>5% reduced	17 (12.3)	48 (37.2)	<0.01
2	1–5% reduced	8 (5.8)	43 (33.3)	<0.01
3	1–5% increased	62 (44.9)	11 (8.5)	<0.01
4	>5% increased	11 (8.0)	4 (3.1)	0.08
5	<1% increased/<1% reduced	40 (29.0)	23 (17.8)	0.02
	**Fasting Glucose**			
1	>25% reduced	2 (1.4)	11 (8.5)	0.007
2	15.1%–25% reduced	7 (5.1)	18 (14.0)	0.013
3	5.1%–15% reduced	18 (13.0)	27 (20.9)	0.08
4	1%–5% reduced	26 (18.8)	17 (13.2)	0.21
5	1%–5% increased	19 (13.8)	6 (4.7)	0.01
6	5.1%–15% increased	18 (13.0)	9 (7.0)	0.10
7	15.1%–25% increased	4 (2.9)	7 (5.4)	0.29
8	>25% increased	8 (5.8)	0 (0.0)	0.005
9	Normal FG after 12-months	30 (21.7)	60 (46.5)	<0.01
10	FG > 7mmol/L after 12-months	19 (13.8)	5 (3.9)	0.005

Note: Data was presented as number of participants (% in respective group). The differences between the groups were tested by Chi-Square test of proportions (Total *n* for GA and IG was 138 and 129, respectively). *p* < 0.05 was considered as significant.

## Supplementary Materials

The following are available online at https://www.mdpi.com/2072-6643/12/2/464/s1, Table S1: Baseline, Anthropometric, and Biochemical characteristics, according to study participants who completed/discontinued the study; Table S2: Baseline characteristics of study participants according to sex; Table S3: Improvements in weight, BMI, waist, hips and HDL-C in female participants’ overtime; Table S4: Improvements in weight, BMI, waist, hips and HDL-C in male participants’ overtime.
