# Supplementary material for: Effects of a 12-Month Intensive Lifestyle Monitoring Program in Predominantly Overweight/Obese Arab Adults with Prediabetes
Source: Nutrients. 2020 Feb 12;12(2):464. doi: 10.3390/nu12020464 (PMC7071332; doi:10.3390/nu12020464)
Supplement: Supplementary file 1 [file nutrients-12-00464-s001.pdf]

Table S1. Baseline, Anthropometric, and Biochemical characteristics, according to study participants who completed/discontinued the study

	Completed Study			Discontinued Study		
Parameters	GA (N=138)	IG (N=129)		GA (N=12)	IG (N=21)	p
Age group (year)^						
20-29	13 (9.4)	12 (9.3)		1 (8.3)	4 (19.0)	
30-39	36 (26.1)	27 (20.9)	0.09	4 (33.3)	2 (9.5)	0.12
40-49	44 (31.9)	60 (46.5)		2 (16.7)	10 (47.6)	
50-73	45 (32.6)	30 (23.3)		5 (41.7)	5 (23.8)	
Sex^						
Male	35 (25.4)	44 (34.1)	0.08	4 (33.3)	5 (23.8)	
Female	103 (74.6)	85 (65.9)		8 (66.7)	16 (76.2)	0.42
Nutritional status^						
Lean	8 (5.8)	14 (10.9)		0 (0.0)	3 (14.3)	
Overweight	32 (23.2)	38 (29.5)	0.22	3 (25.0)	7 (33.3)	0.27
Obese	88 (63.8)	69 (53.5)		8 (66.7)	11 (52.4)	
NA	10 (7.2)	8 (6.2)		1 (8.3)	0 (0.0)	
Weight (kg)#	82.56 ± 13.8	80.71 ± 15.7	0.33	79.15 ± 10.9	80.06 ± 19.6	0.88
BMI (kg/m^2)#	33.13 ± 5.9	31.67 ± 6.0	0.06	31.91 ± 3.3	31.38 ± 6.6	0.80
Waist (cm)#	96.86 ± 8.1	96.62 ± 13.1	0.87	95.65 ± 4.9	95.75 ± 15.2	0.97
Hips (cm)#	111.08 ± 8.6	111.50 ± 11.5	0.76	108.78 ± 2.9	112.35 ± 12.6	0.44
Systolic BP (mm Hg)#	120.28 ± 13.5	121.23 ± 14.6	0.59	117.45 ± 13.3	120.33 ± 16.4	0.62
Diastolic BP (mm Hg)#	76.70 ± 11.8	76.08 ± 10.8	0.67	76.1 ± 8.3	72.19 ± 12.5	0.36
Total Cholestrol (mmol/l)#	4.84 ± 1	5.25 ± 1.2	<0.01	4.71 ± 1.0	4.98 ± 1.0	0.45
HDL-C (mmol/l)#	1.07 ± 0.3	1.17 ± 0.4	0.03	1.04 ± 0.3	1.16 ± 0.4	0.34
Triglyceride (mmol/l)#	1.47 (1.1,2.1)	1.48 (1.1,2.0)	0.75	1.80 (1.2,2.1)	1.64 (1.3,2.1)	0.96
Fasting Glucose (mmol/l)#	6.17 ± 0.6	6.11 ± 0.4	0.35	5.87 ± 0.3	6.12 ± 0.4	0.07
Insulin (μU/ml)$	15.78 (11.9,16.1)	16.56 (16.5,16.7)	<0.01	12.84(9.7,15.9)	16.53 (16.5,16.8)	0.04

Note: Data presented as N (%) for categorical variables (^); Mean±SD for continuous normal variables (#); and medians (25th percentile, 75th percentile) for continuous non-normal variables ($). The difference between groups at baseline was calculated by independent samples t-test and Mann-Whitney U-test for Gaussian and non-Gaussian variables respectively, and χ^2 test for categorical variables. $p<0.05$ is taken as significant. NA, data not available; BP, blood pressure; HDL, high density lipoprotein.

Table S2. Baseline characteristics of study participants according to sex

Parameters	Females			Males		
	GA (N=111)	IG (N=101)		GA (N=39)	IG (N=49)	P
Age group (year)^						
20-29	8 (7.2)	16 (15.8)		6 (15.4)	0 (0.0)	
30-39	31 (27.9)	23 (22.8)	0.04	9 (23.1)	6 (12.2)	0.003
40-49	46 (32.4)	42 (41.6)		10 (25.6)	28 (57.1)	
50-73	46 (32.4)	20 (19.8)		14 (35.9)	15 (30.6)	
Nutritional status^						
Lean	2 (1.8)	11 (10.9)		4 (15.4)	5 (10.2)	
Overweight	23 (20.7)	32 (31.7)	0.005	12 (30.8)	14 (28.6)	0.83
Obese	80 (72.1)	55 (54.5)		16 (41.0)	25 (51.0)	
NA	6 (5.4)	3 (3.0)		7 (12.8)	5 (10.2)	
Weight (kg)#	81.32 ± 13.3	77.55 ± 16.4	0.07	85.26 ± 14.4	87.44 ± 13.7	0.49
BMI (kg/m^2)#	33.93 ± 5.4	31.96 ± 6.4	0.02	30.25 ± 6.3	30.86 ± 5.4	0.65
Waist (cm)#	96.35 ± 7.4	93.07 ± 13.1	0.06	98.07 ± 9.3	104.72 ± 10.2	0.009
Hips (cm)#	112.36 ± 7.5	109.99 ± 11.9	0.12	106.44 ± 9.1	115.59 ± 9.9	<0.01
Systolic BP (mm Hg)#	120.12 ± 14.1	118.39 ± 14.8	0.40	119.81 ± 11.3	127.00 ± 13.2	0.02
Diastolic BP (mm Hg)#	76.92 ± 12.4	74.80 ± 11.6	0.21	75.74 ± 7.9	77.07 ± 9.8	0.53
Total Cholestrol (mmol/l)#	4.88 ± 0.9	5.14 ± 1.1	0.07	4.68 ± 0.9	5.36 ± 1.4	0.01
HDL-C (mmol/l)#	1.07 ± 0.3	1.19 ± 0.4	0.008	1.08 ± 0.4	1.12 ± 0.4	0.68
Triglyceride (mmol/l)#	1.39 (1.1,2.0)	1.43 (1.0,1.8)	0.36	1.73 (1.3,2.1)	1.71 (1.3,2.6)	0.39
Fasting Glucose (mmol/l)#	6.12 ± 0.6	6.12 ± 0.4	0.96	6.21 ± 0.7	6.09 ± 0.4	0.32
Insulin (μU/ml)$	15.72 (10.7,16.2)	16.54 (16.5,16.7)	<0.01	15.79(14.2,16.1)	16.59(16.4,16.8)	0.01

Note: Data presented as N (%) for categorical variables (^); Mean±SD for continuous normal variables (#); and medians (25th percentile, 75th percentile) for continuous non-normal variables ($). The difference between groups at baseline was calculated by independent samples t-test and Mann-Whitney U-test for Gaussian and non-Gaussian variables respectively, and χ^2 test for categorical variables. $p<0.05$ is taken as significant. NA, data not available; BP, blood pressure; HDL, high density lipoprotein.

Table S3. Improvements in weight, BMI, waist, hips and HDL-C in female participants' overtime

	Groups		Intervention Effects (Adjusted Mean change, p)			
Time-point	GA (N=103)	IG (N=85)	GA(6M Vs B)	GA(12M Vs B)	IG(6M Vs B)	IG(12M Vs B)
Anthropometric characteristics						
Weight (kg) #						
Baseline	81.60 ± 13.6	77.81 ± 15.9	0.82, 0.54	0.80,0.54	-1.04,<0.01	-2.78,<0.01
6-months	82.42 ± 14.3	76.77 ± 15.7				
12-months	82.40 ± 13.5	75.18 ± 15.9				
BMI (kg/m²) #						
Baseline	34.03 ± 5.5	32.07 ± 6.3	0.35,0.55	0.37,0.56	-0.41,0.001	-1.13,<0.01
6-months	34.38 ± 5.8	31.66 ± 6.4				
12-months	34.32 ± 5.4	31.01 ± 6.6				
Waist (cm) #						
Baseline	96.43 ± 7.6	93.19 ± 12.8	0.26,0.26	0.20,0.87	0.12,0.51	-1.15,0.06
6-months	95.78 ± 6.3	93.43 ± 13.5				
12-months	95.80 ± 7.1	92.06 ± 12.7				
Hips (cm) #						
Baseline	112.49 ± 7.7	109.88 ± 11.8	0.57,0.51	0.46,0.13	-0.61,0.16	-1.41,0.05
6-months	112.85 ± 7.4	109.24 ± 12.2				
12-months	112.47 ± 6.8	108.32 ± 12.4				
HDL-Cholesterol (mmol/l) #						
Baseline	1.06 ± 0.3	1.20 ± 0.4	-0.12,0.03	-0.13,0.01	0.05,0.30	0.06,0.31
6-months	0.93 ± 0.4	1.26 ± 0.4				
12-months	0.94 ± 0.4	1.25 ± 0.4				

Note: Data presented as Mean ± SD for continuous normal variables (#).The three time-points were baseline (B), 6-months (6M) and 12-months (12M). The intervention affects gives the mean difference within groups at follow-up compared with baseline, and associated p-value; and was calculated by repeated samples t-test. P-value<0.05 was considered as significant.

Table S4. Improvements in weight, BMI, waist, hips and HDL-C in male participants' overtime

	Groups		Intervention Effects (Adjusted Mean change, p)			
Time-point	GA (N=35)	IG (N=44)	GA(6M Vs B)	GA(12M Vs B)	IG(6M Vs B)	IG(12M Vs B)
Anthropometric characteristics						
Weight (kg) #						
Baseline	85.55 ± 14.5	86.81 ± 13.4				
6-months	85.17 ± 13.5	85.44 ± 13.9	-0.38, 0.58	0.34,0.96	-1.34,<0.01	-3.20,<0.01
12-months	86.05 ± 14.0	83.81 ± 13.9				
BMI (kg/m^2) #						
Baseline	30.29 ± 6.6	30.82 ± 5.3				
6-months	30.15 ± 6.2	30.35 ± 5.6	-0.14,0.59	0.10,0.94	-0.46,<0.01	-1.09,<0.01
12-months	30.41 ± 6.5	29.68 ± 5.7				
Waist (cm) #						
Baseline	98.26 ± 9.4	104.26 ± 10.5				
6-months	97.06 ± 8.8	104.57 ± 10.6	-0.43,0.56	0.09,0.67	0.21,0.66	0.18,0.82
12-months	98.35 ± 7.9	104.51 ± 10.1				
Hips (cm) #						
Baseline	106.49 ± 9.6	115.11 ± 9.9				
6-months	105.17 ± 7.8	114.33 ± 9.1	0.43,0.61	0.31,0.33	-1.01,0.12	-0.97,0.07
12-months	105.82 ± 7.5	114.60 ± 9.7				
HDL-Cholesterol (mmol/l) #						
Baseline	1.10 ± 0.4	1.10 ± 0.4				
6-months	0.98 ± 0.5	0.98 ± 0.4	-0.15,0.04	0.06,0.43	-0.10,0.14	-0.16,0.09
12-months	1.06 ± 0.5	0.89 ± 0.2				

Note: Data presented as Mean ± SD for continuous normal variables (#).The three time-points were baseline (B), 6-months (6M) and 12-months (12M). The intervention affects gives the mean difference within groups at follow-up compared with baseline, and associated p-value; and was calculated by repeated samples t-test. P-value<0.05 was considered as significant.
